# Cine Computed Tomography Angiography Evaluation of Blood Flow for Full Face Transplant Surgical Planning

**Published:** 2012-12-18

**Authors:** Geoffroy C. Sisk, Kanako K. Kumamaru, Kurt Schultz, Ericka M. Bueno, J. Rodrigo Diaz-Siso, Elizabeth George, Marta M. Redjaee, Dimitrios Mitsouras, Michael L. Steigner, Bohdan Pomahac, Frank J. Rybicki

**Affiliations:** ^a^Division of Plastic Surgery, Department of Surgery; ^b^Applied Imaging Science Laboratory, Department of Radiology, Brigham and Women's Hospital, Boston, Mass; ^c^Toshiba Medical Research Institute USA, Vernon Hills, Ill

## Abstract

**Objective:** Screening for full face transplantation candidates includes computed tomographic vascular mapping of the external carotid distribution for potential arterial and venous anastomoses. The purpose of this study is to illustrate the benefits and drawbacks of cine computed tomographic imaging for preoperative vascular mapping compared with best arterial and venous phase static images. **Methods:** Two image data sets were retrospectively created and compared for diagnostic findings. The first set of images was the clinical cine computed tomographic acquisition including all phases. The second set of images was composed of the best arterial and best venous phases extracted from the cine loop and determined by the quality of contrast enhancement. For each patient, the benefits and drawbacks of the cine loop were documented in consensus by a plastic surgeon and a radiologist. **Results:** Cine loop analysis identified retrograde arterial filling not illustrated on the static images alone. Cine assessment identified most of the major vessels necessary for surgery, whereas the static images depicted small vessels more clearly, particularly in the crowded vessel takeoffs. **Conclusions:** Cine computed tomographic images provide data on direction of blood flow, which is important for preoperative planning. Combination of cine computed tomographic and the best static images will allow comprehensive vascular assessment necessary for future successful full face transplantation.

Full face transplantation for eligible patients^1^ addresses substantial deficits in structure and function of the face that cannot be satisfactorily treated with reconstructive surgery. Outcomes are highly encouraging,^2^ with aesthetic and functional advantages over conventional reconstructive procedures such as multistaged free flaps.^3^

Standard-of-care vascular screening for face transplant candidates uses wide-area detector^4^^-^6 axial computed tomography (CT) angiography to identify the location, caliber, and course of vessels best suited for the anastomoses to the facial allograft vasculature.^7^^-^9 State of the art acquisitions^10^^,^^11^ also create dynamic angiograms, or cine loops, that illustrate dynamic blood flow that is important for surgical planning. To date, the benefits and drawbacks of cine mode for transplant candidates have not been systematically studied. The purpose of this study was to illustrate dynamic vascular flow for full face transplantation surgical planning and to subjectively compare the cine images with the optimal static images in arterial and venous phases.

## METHODS

### Subjects

Three previously reported full face transplant candidates^2^ signed written informed consent approved by our Institutional Human Research Committee, voluntarily enrolled in clinical trial NCT01281267, and are documented in the US Army Medical Research and Materiel Command's Human Research Protection Office.

In brief, subject 1 was a 25-year-old man who suffered burns secondary to high-voltage injury to the face and scalp and subsequently underwent conventional reconstruction with multiple free flaps and split-thickness skin grafts. Subject 2 was a 30-year-old man after a motor vehicle accident and high-voltage injury to the face. Subject 3 was a 57-year-old woman who was left disfigured by a chimpanzee attack, suffering substantial damage to her central face and requiring multiple surgical reconstruction attempts.

### CT imaging

The CT protocol has been previously described.^7^ Regarding the 320-detector row scanning (Aquilion One; Toshiba Medical Systems, Tochigi-ken, Japan), axial imaging over time spanning up to 16 cm in craniocaudal coverage after intravenous contrast (iopamidol 370 mg of iodine/mL, Isovue-370; Bracco Diagnostics, Princeton, NJ) was power injected (Empower CTA; Acist Medical, New York, NY) at contrast flow rates of 6 mL per second. For each subject, 21 to 24 volumes were acquired in Digital Imaging and Communications in Medicine format.

### Image postprocessing

For each subject, 1- to 2-mm maximum intensity projection cine angiography^12^^,^^13^ was reformatted using video software designed for use at the CT console, plus postprocessing via a dedicated image postprocessing workstation (Vitrea, Version 6.1; Vital Images, Minnetonka, Minn). All visualized vessels were segmented using semiautomated methods supplemented by manual tracings. The best static arterial and venous phases were extracted from the cine loop, as determined by the quality of contrast enhancement.

### Image interpretation

The cine and best static image sets were retrospectively compared for diagnostic findings by the consensus reading from 2 experienced readers (a plastic surgeon and a radiologist). For each patient, the clinical benefits and drawbacks of the cine, when compared with the best static imaging data sets, were documented. Since display in standard (axial, coronal, and sagittal) anatomic planes does not adequately depict the complex relationship between structures, arbitrary cut planes were used for the assessment.

## RESULTS

The total imaging time was less than 45 minutes for all 3 subjects. All images were acquired with less than 100 mL of contrast material, and the estimated, effective total radiation dose was less than 10 mSv for all subjects.

### Arterial anatomy

#### Subject 1

**Figure 1:**
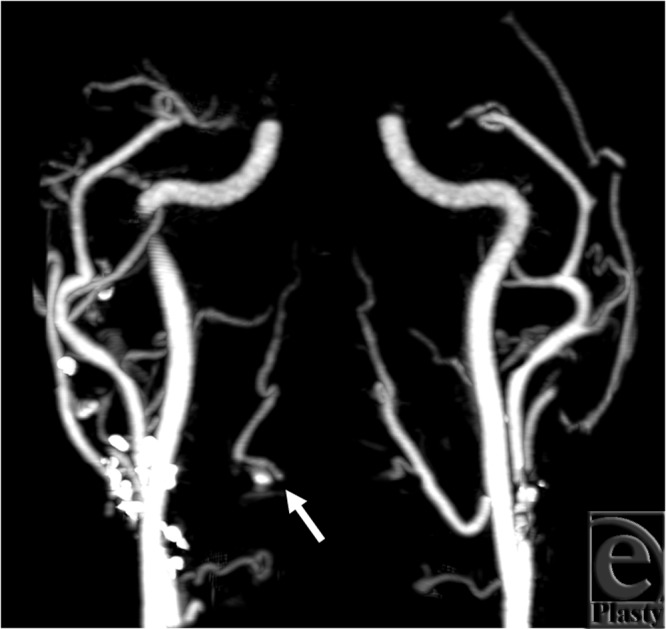
Best arterial phase from subject 1. The right lingual artery is illustrated in only distal parts (arrow).

The cine loop (see Movie 1

) demonstrates filling of the right lingual artery slightly after the filling of the left side, indicating retrograde flow from the contralateral circulation. On the static image, the proximal right lingual artery is not visualized; only distal portions of the artery fill with contrast, making it difficult to determine whether there is a joint facial-lingual segment that branches from the external carotid artery, or rather that this vessel fills in a retrograde fashion (Fig 1). Other major arterial findings are consistent between cine and static image analyses.

#### Subject 2

The findings from the cine and best arterial static images were consistent; all major vessels appear widely patent and follow their natural courses into the injured tissues (see Movie 2

).

#### Subject 3

**Figure 2:**
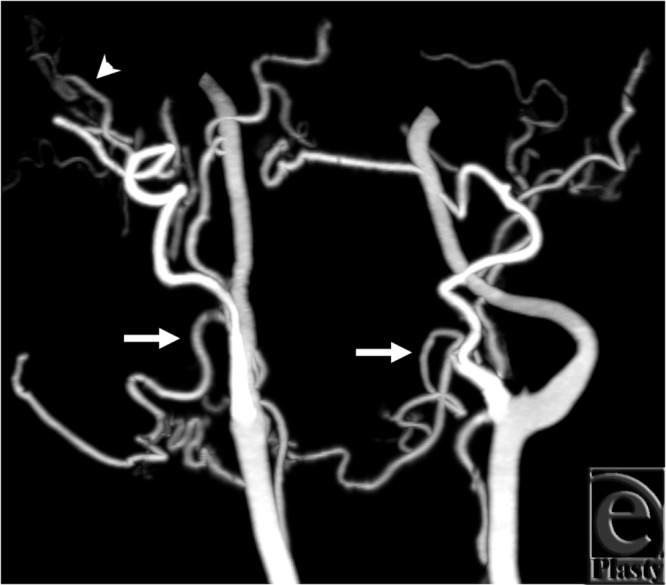
Best arterial phase from subject 3. Facial arteries (arrows) are well opacified and look normal on both sides. The right superficial temporal artery (arrowhead) is also well depicted.

The cine images (see Movie 3

) demonstrate delayed filling of the left facial artery territory, suggesting some retrograde flow, particularly in the setting of a prominent right facial artery. The arterial phase static image with best enhancement shows a fully opacified facial and lingual artery, without signs of retrograde flow (Fig 2). The right superficial temporal artery was hard to identify in the cine loop because of larger vessels obscuring it, whereas the static image illustrated the superficial temporal artery as the terminal branch of the external carotid artery (Fig 2).

### Venous anatomy

**Figure 3:**
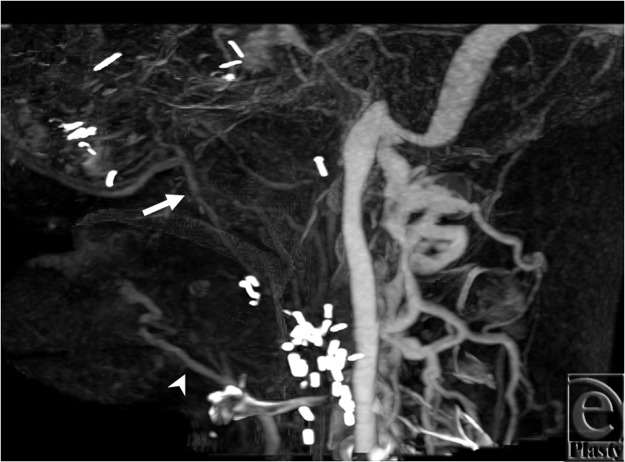
Best venous phase on the right side from subject 1. Facial vein (arrow) is small, ill-defined structure that appears to enter the internal jugular veins independently at the level of the mandible. The lingual vein (arrowhead) appears to drain directly into the anterior jugular vein, which does not communicate with the internal jugular vein.

**Figure 4:**
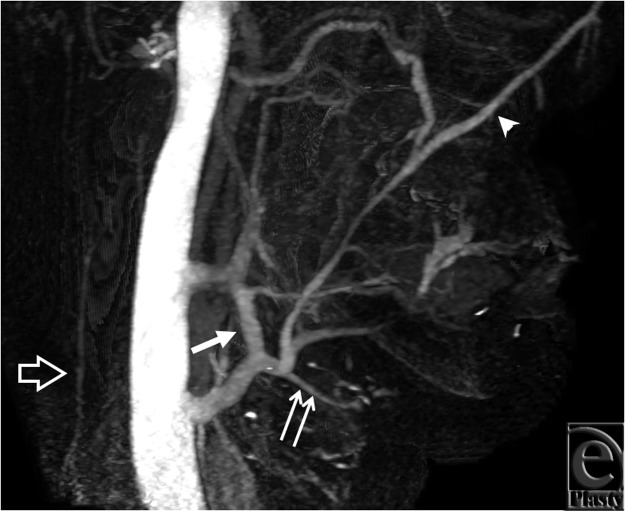
Best venous phase on the right side from subject 2. The retromandibular vein (arrow) is very clearly seen and forms the expected common trunk with the facial vein (arrowhead) before joining the internal jugular vein. The lingual vein (double arrow) is also identifiable. The external jugular vein (open arrow) is seen as a relatively small vessel.

For all 3 subjects, cine image analysis was capable of identifying large veins, namely internal and external jugular, anterior jugular, and the major confluences of facial veins (see Movies 1-3) that are relevant to major reconstructive surgery for anastomosis. In injured tissues that demonstrate substantial collateralization, however, webs of collateralized veins appeared as a thin sheet of contrast, making it difficult to identify small veins and their detailed anatomy. In this regard, static images allow more direct viewing of the trajectories of individual vein segments and their precise paths (Figs 3 and 4).

## DISCUSSION

Candidates for face transplantation require presurgical planning that includes mapping of the vessels being considered for anastomoses to the donor allograft vasculature. As transplantation expands from partial^3^^,^^14^^-^16 to full face procedures, the complexity increases, placing greater demands on vascular mapping. This can be accomplished using CT that provides a comprehensive assessment of the vascular anatomy with the injection of iodinated contrast. In our experience, the overall assessment is complicated by anatomical alterations from the patients’ injuries, early reconstructive procedures, or both.

This study demonstrates the value of cine loop CT for this clinical indication. Earlier, less complex reformations of these data have been used to measure vascular transit times.^9^ This method provides flow direction; these data are invaluable with regard to the precise preoperative planning and the successful transplantation. Furthermore, cine CT should be considered in other complex surgical procedures. Cine images also provides much of the same anatomical information that static images do, whereas static images identify small vessels more easily, particularly in light of the crowded vessel takeoffs seen in subject 2's anatomy.

Noninvasive cine angiography was initially developed using sophisticated magnetic resonance methods.^17^^,^^18^ However in comparison with CT, magnetic resonance methods suffer from greater artifacts largely related to susceptibility from implanted metal used in initial reconstructive attempts.^8^ For these patients, the metal artifacts are generally less for CT.

As the technique and surgical expertise for face transplantation developed and began to demonstrate unrivaled functional, aesthetic, and psychosocial outcomes, wide-area detector CT methods were introduced and enabled cine imaging where each frame in the cine loop was acquired instantaneously and over a volume up to 16 cm in the craniocaudal dimension.^4^

Risks of CT imaging can be separated into those related to contrast administration^19^^,^^20^ and those related to radiation. Since face transplant candidates typically have preserved renal function, in our experience the administration of iodinated contrast has not been problematic. In keeping with other applications,^21^ it is probable that a reduced iodine load would not dramatically impact image quality. With respect to radiation, a cine CT image has greater exposure than static images, owing to the multiple acquisitions over time. There are 2 at-risk organs, the thyroid gland^22^^,^^23^ and the orbits. The cumulative dose to the thyroid should be monitored, and it is also important to limit the radiation to the globes for patients with at least partial vision to avoid cataract formation.^24^

There are several limitations to our study. First, we acknowledge the small patient cohort. However, as the procedure becomes more available, future studies will include more subjects. Second, we did not correlate the imaging findings with the findings at surgery, as it is beyond the scope of this work. Future work will investigate the overall vascular correlation between surgery and computed tomography.

In summary, the cine CT assessment gives information on blood flow that is difficult to obtain from traditional static CT images only. The cine CT, combined with the static images, would provide comprehensive preoperative vascular mapping necessary for full face transplantation. Future studies will evaluate the preoperative findings from these CT images in relation to the posttransplant vascular flow.

## References

[1] Pomahac B, Nowinski D, Diaz-Siso JR (2011). Face transplantation. Curr Probl Surg.

[2] Pomahac B, Pribaz J, Eriksson E (2012). Three patients with full face transplantation. New Engl J Med.

[3] Pomahac B, Pribaz J, Eriksson E (2011). Restoration of facial form and function after severe disfigurement from
burn injury by a composite facial allograft. Am J Transplant.

[4] Rybicki FJ, Otero HJ, Steigner ML (2008). Initial evaluation of coronary images from 320-detector row computed
tomography. Int J Cardiovasc Imaging.

[5] Otero HJ, Steigner ML, Rybicki FJ (2009). The “post-64” era of coronary CT angiography: understanding new
technology from physical principles. Radiol Clin North Am.

[6] Hsiao EM, Rybicki FJ, Steigner ML (2010). CT coronary angiography: 256-slice and 320-detector row
scanners. Curr Cardiol Rep.

[7] Soga S, Ersoy H, Mitsouras D (2010). Surgical planning for composite tissue allotransplantation of the face
using 320-detector row computed tomography. J Comput Assist Tomogr.

[8] Soga S, Pomahac B, Mitsouras D (2011). Preoperative vascular mapping for facial allotransplantation: four-
dimensional computer tomographic angiography versus magnetic resonance
angiography. Plast Reconstr Surg.

[9] Soga S, Wake N, Bueno EM (2011). Noninvasive vascular images for face transplant surgical
planning. ePlasty.

[10] Steigner ML, Otero HJ, Cai T (2009). Narrowing the phase window width in prospectively ECG-gated single heart
beat 320-detector row coronary CT angiography. Int J Cardiovasc Imaging.

[11] Yahyavi-Firouz-Abadi N, Wynn BL, Rybicki FJ (2009). Steroid-responsive large vessel vasculitis: application of whole-brain
320-detector row dynamic volume CT angiography and perfusion. AJNR Am J Neuroradiol.

[12] Rybicki FJ, Lu M, Fail PS, Daniels M (2006). Utilization of thick (>3 mm) maximum intensity projection images in
coronary CTA interpretation. Emerg Radiol.

[13] Lu MT, Ersoy H, Whitmore AG, Lipton MJ, Rybicki FJ (2007). Reformatted four-chamber and short-axis views of the heart using thin
section (</ = 2 mm) MDCT images. Acad Radiol.

[14] Devauchelle B, Badet L, Lengele B (2006). First human face allograft: early report. Lancet.

[15] Lantieri L, Meningaud J, Grimbert P (2008). Repair of the lower and middle parts of the face by composite tissue
allotransplantation in a patient with massive plexiform neurofibroma: a 1-year follow-up
study. Lancet.

[16] Guo S, Han Y, Zhang X (2008). Human facial allotransplantation: a 2-year follow-up study. Lancet.

[17] Ersoy H, Goldhaber SZ, Cai T (2007). Time-resolved MR angiography: a primary screening examination of patients
with suspected pulmonary embolism and contraindications to administration of iodinated
contrast material. AJR Am J Roentgenol.

[18] Kunishima K, Mori H, Itoh D (2009). Assessment of arteriovenous malformations with 3-Tesla time-resolved,
contrast-enhanced, three-dimensional magnetic resonance angiography. J Neurosurg.

[19] Solomon R. (2005). The role of osmolality in the incidence of contrast-induced nephropathy: a
systematic review of angiographic contrast media in high risk patients. Kidney Int.

[20] Parfrey PS, Griffiths SM, Barrett BJ (1989). Contrast material-induced renal failure in patients with diabetes mellitus,
renal insufficiency, or both. A prospective controlled study. N Engl J Med.

[21] Kumamaru KK, Steigner ML, Soga S (2011). Coronary enhancement for prospective ECG-gated single R-R axial 320-MDCT
angiography: comparison of 60- and 80-mL iopamidol 370 injection. AJR Am J Roentgenol.

[22] Shu KM, MacKenzie JD, Smith JB (2006). Lowering the thyroid dose in screening examinations of the cervical
spine. Emerg Radiol.

[23] Rybicki F, Nawfel RD, Judy PF (2002). Skin and thyroid dosimetry in cervical spine screening: two methods for
evaluation and a comparison between a helical CT and radiographic trauma
series. AJR Am J Roentgenol.

[24] Neriishi K, Nakashima E, Akahoshi M (2012). Radiation dose and cataract surgery incidence in atomic bomb survivors,
1986-2005. Radiology.

